# A behavioral science/behavioral medicine core curriculum proposal for Japanese undergraduate medical education

**DOI:** 10.1186/s13030-015-0051-3

**Published:** 2015-11-24

**Authors:** Akizumi Tsutsumi

**Affiliations:** Kitasato University School of Medicine, 1-15-1 Kitasato, Minami, Sagamihara, Kanagawa 252-0374 Japan

## Abstract

Behavioral science and behavioral medicine have not been systematically taught to Japanese undergraduate medical students. A working group under the auspices of Japanese Society of Behavioral Medicine developed an outcome-oriented curriculum of behavioral science/behavioral medicine through three processes: identifying the curriculum contents, holding a joint symposium with related societies, and defining outcomes and proposing a learning module. The behavioral science/behavioral medicine core curriculum consists of 11 units of lectures and four units of practical study. The working group plans to improve the current core curriculum by devising formative assessment methods so that students can learn and acquire attitude as well as the skills and knowledge necessary for student-centered clinical practice.

## Introduction

The Content Specifications for the Japanese National Medical Practitioners Qualifying Examination, the “*IX. Treatment*” section of Principles of Medicine, and the “*II. Psychiatric and Psychosomatic Disorders*” section of Medicine include a considerable number of terms related to behavioral medicine. Furthermore, while psychology is taught as a liberal arts subject, it is not necessarily compulsory. Therefore, behavioral medicine has not been directly addressed in Japanese medical education [1]. Meanwhile, behavioral science and social medicine are specified as subjects that must be included and implemented within the educational programs provided by Japanese medical schools and medical universities that are preparing to undergo certified evaluation based on the standards of the World Federation for Medical Education (see Table 1) [2].

**Table 1:** Annotations of behavioural and social sciences, medical ethics, and medical jurisprudence (in part) provided in the WFME (World Federation for Medical Education) global standards for quality improvement in basic medical education

*Behavioural and social sciences* would - depending on local needs, interests and traditions - include biostatistics, community medicine, epidemiology, global health, hygiene, medical anthropology, medical psychology, medical sociology, public health and social medicine.
The *behavioural and social sciences, medical ethics and medical jurisprudence* would provide the knowledge, concepts, methods, skills and attitudes necessary for understanding socio-economic, demographic and cultural determinants of causes, distribution and consequences of health problems as well as knowledge about the national health care system and patients’ rights. This would enable analysis of health needs of the community and society, effective communication, clinical decision making and ethical practices.

In Japanese medical education, behavioral science and behavioral medicine are rarely treated as independent curricula and a system for teaching them systematically has not yet been established. Therefore, the Japanese Society of Behavioral Medicine has decided to develop a curriculum for these subjects, which it wishes to recommend for medical education in Japan. In this article, I introduce the background against which the Curriculum was developed, the development process, and the Curriculum itself.

## Background

In 2013, the 19th Annual Meeting of the Japanese Society of Behavioral Medicine was held on the theme of “How to utilize behavioral medicine: From education to clinical practice.” The Meeting featured a Presidential lecture by Professor Koji Tsuboi entitled “Behavioral Medicine and Medical Education.” Then, during the preparations for the 20th Annual Meeting, members discussed the importance of and the Society’s contribution to educational issues, and upon the instruction of Professor Shinobu Nomura, the Chairman of the Board of Directors, established a Working Group for the Development of a Core Curriculum for Behavioral Sciences (hereafter referred to as “the Working Group”) under the Japanese Society of Behavioral Medicine Educational and Training Committee (chaired by Professor Mutsuhiro Nakao). Making use of the Japanese Society of Behavioral Medicine’s interdisciplinary characteristics, the Working Group members were selected from the fields of psychology, social medicine, and clinical medicine from among core members who were also actively involved in other related societies.

Since behavioral science and behavioral medicine are interdisciplinary fields, these subjects are not only studied by trainee doctors but by a wide range of trainee health care providers, including nurses, psychologists, and preventative medicine practitioners. Taking into account the needs arising from international certified evaluation, despite focusing on undergraduate medical education, we have decided to expand the curriculum to other occupational categories based on this initial work.

### The development process

The Curriculum was developed through the following three processes: (1) the curriculum content was identified, (2) a joint symposium with related societies and a workshop were held, and (3) outcomes were defined and a learning module proposed.

1. Identifying the curriculum content
  The Working Group began compiling a list of knowledge and skills (competencies) related to behavioral science and behavioral medicine that medical students would need to acquire before graduating. Initially, the Working Group gathered information from some members of the Japanese Society of Behavioral Medicine on curriculum development and proposals at their respective institutions. Furthermore, it gathered information on the activities of other universities and related societies, including the Japan Society for Medical Education, surveys conducted by the Education and Training Committee of the International Society of Behavioral Medicine, and trends at universities in the United States [3–8]. The proposed competencies were moderated, the terminology was edited, and categorical contradictions were removed and a core list was compiled consisting of 52 competencies to be acquired during undergraduate medical education spanning six larger categories (theory and terminology of psychology; theory of behavior modification, treatment and health guidance methods, social psychology/social medicine-related, application of behavior modification, and others).
  Based on this list, we surveyed 111 members of the Japanese Society of Behavioral Medicine using the Delphi method to consolidate the opinions of experts. The first survey was conducted in late October, 2013. Respondents were asked to choose the degree to which each of the 52 competencies should be acquired by students as a minimum requirement before graduating from medical school. There were three options: (1) students do not need to acquire it, (2) students need to know it, and (3) students need to be able to explain or describe it. Based on the results of first round of opinion gathering and the additional competencies proposed by the respondents, the Working Group selected competencies and revised the categories. Then, in early December, 2013, we conducted a second survey of members who agreed to participate during the first survey. Based on the responses to the second survey, we gathered the competencies with a high proportion of (3)s and (2)s and identified the competencies that students need to acquire before graduating from medical school in Japan [9].
2. The joint symposium with related societies and the education workshop
  In March, 2014, the 20th Annual Meeting of the Japanese Society of Behavioral Medicine was held in Kyoto (President: Professor Takeo Nakayama of Kyoto University Graduate School). At the Meeting, the Working Group reported its activities to date, and in order to obtain a wide range of opinions from experts external to the Japanese Society of Behavioral Medicine, held a symposium entitled “Proposing a Core Curriculum for Behavioral Medicine,” to which representatives from the Japan Academy for Health Behavioral Science and the Japanese Association of Behavioral Science were invited. During the symposium, the interim achievements of the Working Group were outlined, and through discussion with representatives and members of related academic societies, the need to develop education that also incorporates Japan’s unique cultural and social circumstances was confirmed, as well as the need for student-centered lectures and practicals. Then, the content of the symposium was summarized in the subsequent Education Workshop held by the Education and Training Committee. Following the 20th Annual Meeting, the Working Group formulated an action plan for the development of a tangible model curriculum based on the competencies identified through the above process.
3. Defining outcomes and proposing a learning module
  While referring to curricula in use at each medical school and graduate school of medicine, we proposed a learning module (a series of model lessons). Although we had initially considered developing a ten-hour “standard” version and a five-hour “minimum” version, we eventually decided to create a standard 1 credit = 15 h learning module and leave the decision about the actual number of study hours to each institution. Specifically, the members of the Working Group split into three groups based on the three divisions of the Society of Behavioral Medicine, psychology, social medicine, and clinical medicine. Then, the psychology and social medicine groups arranged the competencies identified using the Delphi method into units of knowledge and theory to be taught while the clinical medicine group examined methods for acquiring skills and basic approaches for applying this knowledge to clinical cases and treatment. Furthermore, we defined attainment targets for each unit of study. A full debate was held using the findings of the respective groups as springboards for discussion and the learning modules were determined while also taking into account the distribution of content among subject areas. While referring to the open opinions obtained in the member survey, we discussed the outcomes that could be achieved by studying the learning module. In addition, we considered which year of study and what teaching methods would be most effective for helping students to acquire content, which ranged from basic to applied.

### Content of the core curriculum for behavioral science/behavioral medicine

We defined the learning outcomes for education in behavioral science and behavioral medicine as shown in Table 2 and developed a core curriculum and learning module to help students achieve these outcomes before graduating from medical school (see Table 3). The Core Curriculum is based on one *koma* (90 min lesson) per unit of study, and in addition to 11 units of lectures, we have included four units of practical study, for which we recommend teaching methods such as tutorial materials and the case method. The decision to include these practical units was prompted by the need for students to develop the skills and basic approaches for considering how to apply the knowledge acquired in lectures to clinical cases and treatments.

**Table 2:** Outcome of the core curriculum for behavioral science/medicine in Japanese undergraduate medical education

1. Acquire basic knowledge of shaping, motivation, stress, and life-span development.
2. Acquire basic theories and methodologies for maintaining and promoting health and basic knowledge of social stress and health.
3. Acquire basic knowledge of the effects of social and cultural factors on health.
4. Understand the role of communication in (1) maintaining and promoting health and (2) acquire methods for promoting it.
5. Acquire theory and actual knowledge related to responses to stress (stress coping and stress management).
6. Develop the ability to devise (1) strategies for treating hypothetical cases of difficult situations and (2) guidance strategies for maintaining and promoting health by applying the above knowledge and theoretical understanding.
7. Develop the ability to motivate and offer guidance to people so that they can behave in a way that will help them to live healthy lives.

**Table 3:** Learning module for the core curriculum in behavioral science/medicine in undergraduate education

Lecture theme	Lecture keywords and training methods	Attainment targets
1. Shaping	• Imprinting • Classical (respondent) conditioning • Operant (instrumental) conditioning • Cognitive learning • Social learning (observation and imitation) • Brain neurotransmitters	(1) Can explain innate and learned behavior (adaptive learning, non-adaptive learning). (2) Can explain respondent conditioning (learning relationships between stimuli) and operant conditioning (learning relationships between responses and results). (3) Can explain social learning (observation and imitation). (4) Can explain the basic neural processes of behavior.
2. Motivation	• Motivation (intrinsic and extrinsic) • Needs • Frustration • Conflict • Adjustment mechanisms • Defense mechanisms	(1) Can outline physiological motives (self-preservation and species-preservation), intrinsic motives (activity, sensitivity, curiosity, manipulation, etc.), and social motives (achievement, affiliation, affection, control, etc.). (2) Can give examples of motivation. (3) Can outline the relationship between needs and frustration/conflict. (4) Can outline adjustment (defense) mechanisms.
3. Stress (psychological)	• Stressors • Stress reactions • Psychological models of stress • Cognitive appraisal • Coping • Life events • Relaxation techniques	(1) Can outline the major stress theories. (2) Can give examples of stressors in life and everyday activities and their effects on health. (3) Can explain psychosocial factors related to the stress-coping process. (4) Can outline stress management techniques.
4. Stress (environmental) and health	• Workplace stress • Other kinds of stress (childhood stress, parenting stress, etc.) • Stress management • Social support	(1 )Can outline the various kinds of stress encountered in the workplace and daily life. (2) Can outline the relationship between stress in the workplace and daily life and health. (3) Can outline stress management techniques based on environmental adjustment.
5. Life-span development	• Mental development • The life cycle • Gene-environment interaction • Life tasks	(1) Can outline the principles of mental development. (2) Can outline life tasks and the characteristics of mental development at each stage of the life cycle. (3) Can outline genetic factors and environmental factors affecting mental development.
6. Individual differences	• Personality • Type theory • Trait theory • The Big Five • Intelligence • Role theory • Gender	(1) Can outline and compare the approaches of type and trait theories of personality. (2) Can outline personality formation and function. (3) Can outline changes in intelligence and development over time. (4) Can outline role theory. (5) Can outline gender formation.
7. Interpersonal relationships	• Interpersonal cognition • Needs and conflict • Group dynamics • Social adjustment • Interpersonal communication • Culture	(1) Can outline psychological factors related to interpersonal relationships. (2) Can outline the relationship between needs and behavior in interpersonal relationships. (3) Can outline the major interpersonal behaviors (aggressive behavior, helping behavior, etc.). (4) Can outline interpersonal behaviors within groups (competition and cooperation, conformity, obedience and resistance, leadership, etc.). (5) Understands effective interpersonal communication. (6) Can give examples of cultural effects on individuals and groups.
8. Theories of behavior modification	• Motivation • Behavior therapy • Cognitive behavior therapy • Stimulus control • Self-efficacy • Multiple-theory integration model • Empowerment	(1) Can outline motivation through health behavior and behavior modification.
9. Behavior modification techniques	• Lifestyle guidance • Health guidance (smoking cessation/medication counseling) • Teaching and coaching	(1) Can outline the behavioral science treatments applied to clinical medicine.
10. Health communication	• The spread of public health and medical information (guidelines, participation rate for medical examinations) •Doctor-patient communication •Communication between medical practitioners	(1) Can explain how medical information is transmitted to ordinary citizens and patients. (2) Understands how to transmit medical information to ordinary citizens and patients. (3) Can outline problems that occur between medical practitioners (e.g., medical safety problems that arise when the transmission of information is insufficient). (4) Understands techniques for communication between medical practitioners.
11. Society and health	•Inequality and health •Social capital •Social participation •Social epidemiology •Social determinants of health •Cultural competence	(1) Can outline the effects of social conditions and social life on people’s health. (2) Understands the importance of developing cultural competence. (3) Can outline public health strategies.
12 – 15. Seminars/practicums	Proposals for actual treatment strategies and role-plays based on medical scenarios	(1) Can communicate in a way that responds to patients’ psychological needs in various medical settings. (2) Can give clear explanations to patients and can explain the principles of support for helping patients to make decisions on their own. (3) Can teach and support simulated patients based on principles in a way that promotes behavior modification. (4) Can behave in a professional manner and communicate effectively when conveying “bad news” to patients.

We chose to leave decisions regarding the integration of units and the adjustment of the number of *koma* to the respective institutions. For example, it is possible to select lecture content based on the characteristics of the course, for example when using the curriculum for the psychology division of liberal arts courses. In addition, in terms of methods of taking classes, it is possible to teach the basic theory in the lower years of study and have students learn about, for example, the application of behavior modification theory during the upper years of study as part of the Clinical Clerkship or Social Medicine Clerkship courses.

### Future directions: toward the expansion of the curriculum for behavioral medicine

The curricula of medical education are also renewed over time in accordance with changes in social conditions, including advances in medical technology. In the United States, Social and Behavioral Science is a compulsory subject in the unified curriculum that was recently introduced at graduate schools of public health, and graduate schools have begun reorganizing their curricula accordingly. This Core Curriculum was developed by organizing the minimum competencies that Japanese medical students are expected to acquire before graduating from medical school based on the opinions of experts from the Japanese Society of Behavioral Medicine. However, the competencies required of medical students will also change in accordance with the demands of society. The curriculum includes content related to cultural phenomena, a relatively new issue that has received considerable attention recently. Although this content was not included as an independent unit, it is expected that the importance of cultural competence will continue to increase [10]. Therefore, we hope to continue this discussion when revising the curriculum in the future.

We are continuing to discuss the scope of behavioral science and behavioral medicine. Although topics concerning basic interaction between doctors and patients, such as adherence, and the application of behavioral science techniques, such as behavior modification, can be defined as preventative intervention, they are also closely related to clinical practice. In North America, behavioral medicine has been incorporated into general education topics [11] and a significant number of specialist clinical competencies are included in the content specifications for the Behavioral Science section of the United States Medical Licensing Examination [12]. Meanwhile, at the biannual International Congress of Behavioral Medicine meeting, the scope of basic biobehavioral research has expanded rapidly [13]. Recognizing that the overlap between behavioral science and behavioral medicine is, at present, difficult to remove, the Working Group decided to integrate the two fields of study.

In contemporary medical education, students are required to go beyond simply “knowing” by developing their ability to “apply” knowledge. Therefore, problem-based learning (PBL) [14] and team-based learning (TBL) [15], in which students work together in small groups to solve problems concerning hypothetical clinical cases, have been recommended as effective approaches. To help students achieve the outcomes specified in the Curriculum, we recommend the use of practicals and seminars that include activities such as role-plays, for developing holistic understanding of disorders, and practical skills for behavior modification, as well as PBL and TBL methods, which encourage students to consider actual treatment strategies in clinical case scenarios. The Working Group recognizes the importance of developing model case scenarios and commentaries in achieving this goal and has proposed a series of case scenario materials that can be used in the PBL classroom (see Table 4).

**Table 4:** Medical case scenarios for problem-based learning (PBL)

1. High blood pressure case with poor medication adherence (including smoking cessation guidance)
2. Diabetic patient who desires treatment but has poor glycemic control
3. Patient who refuses hospitalization and outpatient care after being diagnosed with cancer
4. Eating disorder patient with poor disease awareness who resists treatment
5. Hyperlipidemic patient with high blood pressure who was severely reprimanded by his/her superior and is unable to go to work due to palpitations, chest oppression, and cold sweats

In the future, it will be necessary to devise and establish new assessment methods in response to the forthcoming expansion of student-centered clinical practice. For example, it is thought that, in addition to skills-based training, students can strengthen their communication skills by receiving feedback on their approaches and attitudes, not only through role-plays but also by interacting with a large number of patients in authentic clinical settings. In addition to summative assessment, it is important to enhance formative assessment methods.

## Conclusions

I hope that medical educators will find this report to be a useful material that forms a basis for developing a comprehensive curriculum in behavioral medicine in Japanese undergraduate medical education. As behavioral medicine and psychosomatic medicine share lots of common elements not only for teaching medical students but also for caring for patients, I believe this report is beneficial to the readers in the field of psychosomatic medicine.
